# Role of Respiration in Mind-Body Practices: Concepts from Contemporary Science and Traditional Yoga Texts

**DOI:** 10.3389/fpsyt.2014.00167

**Published:** 2014-11-25

**Authors:** Shirley Telles, Nilkamal Singh, Acharya Balkrishna

**Affiliations:** ^1^Patanjali Research Foundation, Haridwar, India

**Keywords:** ancient yoga texts, psychosomatic disease, respiratory regulation, affect, relaxation, inner awareness

The concept that interactions between the mind and body influence physical health has existed for a long time (1). These interactions form the basis for mind–body practices. They are considered bi-directional (“top-down” and “bottom-up”), with connections between the brain and peripheral tissues (2). Top-down mechanisms are initiated by the cerebral cortex and include clinical hypnosis, imagery, meditation, and breath awareness. Bottom-up mechanisms on the other hand, stimulate various somato-sensory, viscero-sensory, and chemo-sensory receptors (3, 4), which influence ascending pathways from the periphery to the brainstem and cerebral cortex.

Mind-body practices are believed to act at multiple levels from gene expression at the cellular level to interaction between central brain regions (2). Taylor et al. (2) have proposed four mechanisms: (a) reorganization of cortical and subcortical structures and better interhemispheric balance; (b) improved central regulation of autonomic and immune functions; (c) re-patterning of primary interoceptive and higher order homeostatic mechanisms; and (d) modulation of epigenetic factors such as growth factors or hormones.

These four effects are achieved by different ways including mental imagery, somatic relaxation, and deep breathing. Based on these effects, most mind–body practices are used in the treatment of psychosomatic illness. The ancient Indian practice of yoga is a mind–body practice. According to yoga texts, it is important to understand the origin of psychosomatic illness in order to treat it (*Taittiriya Upanishad*, *circa* 1200 B.C^1^) (5). This text continues to state that mental conflict, especially conflict between the intellect (called the *vignanamaya kosha* in Sanskrit) and the instinct (called the *manomaya kosha* in Sanskrit) causes a disturbance in the subtle, life energy, called *prana* in Sanskrit (5). This concept of an imbalance in the subtle life energy is mentioned in traditional descriptions of yoga but is not mentioned in contemporary medicine. Traditional yoga texts also suggest a solution for the imbalance in *prana*, through slow, deep breathing [*Hatha Yoga Pradipika*, c*irca* 300 A.D.^1^] (6). It has been said that “when the mental state is disturbed, the life energy (*prana*) gets unbalanced and this leads to irregular breath; hence to regulate the mental state the yoga practitioner should regulate the breath” [*Hatha Yoga Pradipika*, *circa* 300 A.D.; Chapter 2, Verse 16^1^] (6).

Yoga regulated breathing acts as both a top-down and bottom-up mind-body practice. There is sufficient neuroanatomical evidence to support the idea that apart from the metabolic (chemoreceptor based) regulation of breathing internal and external factors influence breathing, which has been called behavioral breathing (7). Connections between cortical regions and brainstem respiratory neurons indicate that influences from higher centers can modify metabolic breathing (8, 9). A functional magnetic resonance study in normal subjects who had air hunger (with a low tidal volume) induced by mechanical-ventilation showed increased activity in limbic and paralimbic loci (10). Apart from these central connections, peripheral factors can also influence respiration. Nasal breathing activates olfactory cells; they activate the olfactory bulb and in turn the pre-piriform cortex and piriform cortex (11). Olfactory information ascends directly to limbic areas and hence olfactory information, which can be indirectly connected to breathing, influences emotions.

Apart from being slow, deep and diaphragmatic, yoga breathing includes awareness of the movement of air in the nasal passages (12). Subjective awareness of inner sensations is referred to as interoception (13). An imaging study matched individuals’ subjective perception of their heartbeat with psychometric measures of their interoceptive awareness and emotionality (14). Their findings showed that the right anterior insula (rAI) is important for explicit subjective awareness.

Conventional physiology has found benefits of deep breathing supporting the importance given to regulating the breath in yoga. Slow breathing had a balancing effect on the autonomic nervous system via enhanced parasympathetic activation (15). Slow and deep breathing stimulates inhibitory signals induced by stretch and hyperpolarizes cells, leading to synchronization of neural elements in the heart, lungs, limbic system, and cortex (16). Slow breathing also enhances vagal activity leading to reduced psychophysiological arousal, decreased sympathetic activity, and stress responses (17). Other effects include an increase in the antioxidant levels and hence deep breathing may help to reduce oxidative stress (18). In addition, deep breathing has been found to decrease cortisol and increase melatonin (19), possibly by the effects on the hypothalamic neuroendocrine regulation (15).

## Summary

Psychosomatic illness is often successfully managed by mind–body practices. Contemporary medicine believes that mental conflict contributes to psychosomatic illness. Yoga is a traditional Indian mind–body practice, which also attributes psychosomatic illness to mental conflict. Traditional yoga texts describe that this conflict causes an imbalance in the subtle life energy, called *prana*, which is not recognized by contemporary medicine. Yoga texts also mention a solution for it, in the form of deep breathing. The beneficial effects of deep breathing are supported by contemporary science.

## Conflict of Interest Statement

The authors declare that the research was conducted in the absence of any commercial or financial relationships that could be construed as a potential conflict of interest.

## Footnotes

^1^The citations for the traditional yoga texts are commentaries (in English) as the original texts are written in Sanskrit.
